# Hierarchical disentanglement of contextual from compositional risk factors of diarrhoea among under-five children in low- and middle-income countries

**DOI:** 10.1038/s41598-021-87889-2

**Published:** 2021-04-20

**Authors:** Adeniyi Francis Fagbamigbe, A. Olalekan Uthman, Latifat Ibisomi

**Affiliations:** 1 Department of Epidemiology and Medical Statistics, College of Medicine, University of Ibadan, Ibadan, Nigeria; 2 Division of Health Sciences, Populations, Evidence and Technologies Group, University of Warwick, Warwick, UK; 3 Health Data Science Group, Division of Population and Behavioural Sciences, School of Medicine, University of St Andrews, Fife, UK; 4 School of Public Health, University of the Witwatersrand, Johannesburg, South Africa; 5 Nigerian Institute of Medical Research, Lagos, Nigeria

**Keywords:** Statistics, Risk factors, Epidemiology, Public health, Epidemiology

## Abstract

Several studies have documented the burden and risk factors associated with diarrhoea in low and middle-income countries (LMIC). To the best of our knowledge, the contextual and compositional factors associated with diarrhoea across LMIC were poorly operationalized, explored and understood in these studies. We investigated multilevel risk factors associated with diarrhoea among under-five children in LMIC. We analysed diarrhoea-related information of 796,150 under-five children (Level 1) nested within 63,378 neighbourhoods (Level 2) from 57 LMIC (Level 3) using the latest data from cross-sectional and nationally representative Demographic Health Survey conducted between 2010 and 2018. We used multivariable hierarchical Bayesian logistic regression models for data analysis. The overall prevalence of diarrhoea was 14.4% (95% confidence interval 14.2–14.7) ranging from 3.8% in Armenia to 31.4% in Yemen. The odds of diarrhoea was highest among male children, infants, having small birth weights, households in poorer wealth quintiles, children whose mothers had only primary education, and children who had no access to media. Children from neighbourhoods with high illiteracy [adjusted odds ratio (aOR) = 1.07, 95% credible interval (CrI) 1.04–1.10] rates were more likely to have diarrhoea. At the country-level, the odds of diarrhoea nearly doubled (aOR = 1.88, 95% CrI 1.23–2.83) and tripled (aOR = 2.66, 95% CrI 1.65–3.89) among children from countries with middle and lowest human development index respectively. Diarrhoea remains a major health challenge among under-five children in most LMIC. We identified diverse individual-level, community-level and national-level factors associated with the development of diarrhoea among under-five children in these countries and disentangled the associated contextual risk factors from the compositional risk factors. Our findings underscore the need to revitalize existing policies on child and maternal health and implement interventions to prevent diarrhoea at the individual-, community- and societal-levels. The current study showed how the drive to the attainment of SDGs 1, 2, 4, 6 and 10 will enhance the attainment of SDG 3.

## Introduction

Despite killing over 2000 children every day—more than AIDS, malaria, and measles combined, diarrhoea received less attention from child health programmers and funders compared with other child killer diseases^1,2^. In 2015, there were an estimated 2.3 billion diarrhoea-related illnesses of which 1.3 million deaths occurred globally with about half a million diarrhoea-related deaths among under-five children annually^3,4^. Under-five mortality is a core indicator of the overall wellbeing of a society and a measure of the progress made by societies in healthcare system and management^5,6^. In 2015, there were nearly 6 million under-five mortalities including 45% neonatal deaths^7^ of which about 90% were in sub-Saharan Africa and South Asia^8^. The disproportionate ratio of 73.1–5.3 under-five deaths per 1000 live births in the low-income countries and high-income countries respectively is a source of concern^9^. Effective interventions such as prevention and control of childhood diseases, enhanced health care programmes, effective and efficient provision and utilization of health care facilities including adequate and timely immunization and vaccination are very essential to reduce under-five mortalities^5,6^.

Although remarkable progress has been reported in the reduction of under-five mortality and morbidities globally^8,10–12^, childhood diarrhoea remains major public, clinical and social health challenges in the Low- and Middle-Income Countries (LMIC)^3^. There have been differences but a close ranking of diarrhoea burden among these countries in the literature. While Gill et al. reported that diarrhoea disease is the 9th leading cause of death globally but 4th among children under-5 years^13^, the ranks were 8th and 5th respectively in a Global Burden of Disease (GBD) study^14^. About nine of every annual global diarrhoea-related deaths among under-five children occurs in Sub-Saharan Africa and South Asia^3,15^. Worse still, the survivors of diarrhoea are faced with a long-time higher risk of growth faltering, ill health, stunting, and cognitive impairment^16,17^.

Diarrhoea diseases have continued to cause monumental morbidity and mortality in developing countries^3^. With a reported 15% of global deaths among children under-5 years attributable to diarrhoea^4,10,11,15^, it remains one of the topmost children killers in LMIC^8,10,12,18–21^. The findings of Mokomane et al. corroborated the GBD and WHO assertions that acute diarrhoea disease is one of the topmost causes of global morbidity and mortality particularly among young children in resource-constrained countries^17^. The burden of diarrhoea is much higher in LMIC than in the high-income countries^10,14,22,23^ with worst hits in the South Asia and sub-Saharan Africa regions, both having 52% of all the burden^24^. This is quite plausible as these regions constitute the majority of LMIC. Also, researchers were unanimous that diarrhoea is caused by a diverse range of aetiological agents, inclusive of bacterial enteritis which is very common in LMIC^16,17,25^.

Global efforts have been made to strengthen health systems in every country to offer interventions that could prevent diarrhoea and save the lives of several millions of children. Among several efforts to combat diarrhoea is the pronouncement of the Sustainable Development Goal (SDG)-3 which focused on ensuring healthy lives and promoting the well-being of all, with specific target 3.2 to end preventable deaths of children under 5 years of age by 2030, and drastic reduction of neonatal and under-five mortalities to at least as low as 12 and 25 per 1000 live births respectively in all countries^26^. Also, a study coordinated by WHO and UNICEF, developed the Global Action Plan for the Prevention and Control of Pneumonia and Diarrhoea^27^. The group established plans to reduce the severe incidence and deaths due to diarrhoea in children by 2025^14^. Several other national and local interventions have been made in different countries to alter the tides in diarrhoea^10,18^. The central aim of these interventions is to increase life expectancy with a reduction in widespread diseases associated with early mortality^6^.

These efforts notwithstanding, the incidence of diarrhoea and diarrhoea-related under-five mortality remains a major challenge in most LMIC. It is worth noting that although improvements in the standard of living, advances in sanitation, water treatment and food safety awareness have brought about a reduction in the total global deaths due to diarrhoea, the morbidity from diarrhoea have remained exceptionally high and has accounted for substantial economic and societal losses^17^. Of greater concern is that there may be a rebound in the upsurge of diarrhoea-associated mortality in the nearest future due to increasing diarrhoea-related morbidity, urbanization, global warming and climate change^17,28,29^. Besides, prolonged episodes of diarrhoea have been linked to significant comorbidities and has put children at risk of a vicious cycle of diarrhoea and malnutrition^30^.

There is, therefore, an urgent need to assess the risk factors central to most LMIC as a critical step in the potential reworking of intervention strategies to reduce the incidence of diarrhoea among under-five children. Literature is replete with the fact that WASH factors (presence of a domesticated animal in the house, having animal shed in proximity to the household, use of cow dung in household and open field defecation), maternal age and education, household wealth quintile, child age, sex, birth weight, birth order, household wealth quintile and location of the resident are associated with the experience of diarrhoea among under-five children^10,17,20,31,32^. These studies have established a pathway between the factors and diarrhoea diseases. This study, therefore, reported the prevalence of diarrhoea among under-5 year children in 57 LMIC. The study also identified the individual-specific factors, neighbourhood factors and country-level factors that affect the occurrence of diarrhoea among under-five children in the 57 LMIC using hierarchical Bayesian logistic regression models.

## Methods

### Study design and data

The cross-sectional and nationally representative Demographic and Health Surveys (DHS) data collected during household surveys across most LMIC were used for this study. We extracted and pooled the latest recoded “children data” from the DHS that collected information on diarrhoea, conducted between 2010 and 2018 and available in the DHS data domain by March 2019. Only 57 LMIC met these criteria and were included in this study. The DHS uses a multi-stage, stratified sampling design with households as the sampling unit^33,34^. However, due to differences in the administrative levels in different countries, the number of sampling stages differed. Country-specific sampling methodologies are available at dhsprogram.com and in the country-specific reports^35–37^. Sampling weights were computed and provided alongside the data from each country by DHS and were applied to our analysis. The sampling weights were based on the multi-stage sampling procedures to ensure representation of the general population. All the DHS questionnaires were standardized and implemented across all countries with similar interviewer training, supervision, and implementation protocols.

### Data source

The secondary data used for this study is available on request from the owners of the data at https://www.dhsprogram.com/data/dataset_admin/login_main.cfm.

### Dependent variable

Our dependent variable is diarrhoea. Firstly, women were asked to name all births they had within 5 years before the survey dates. They were then asked if any of the children had at least an episode of diarrhoea within 2 weeks preceding the survey date. The response is binary with children who had diarrhoea coded as “1” and “0” otherwise.

### Independent variables

We used three categories of explanatory variables.

#### Individual-level factors

**Figure 1:**
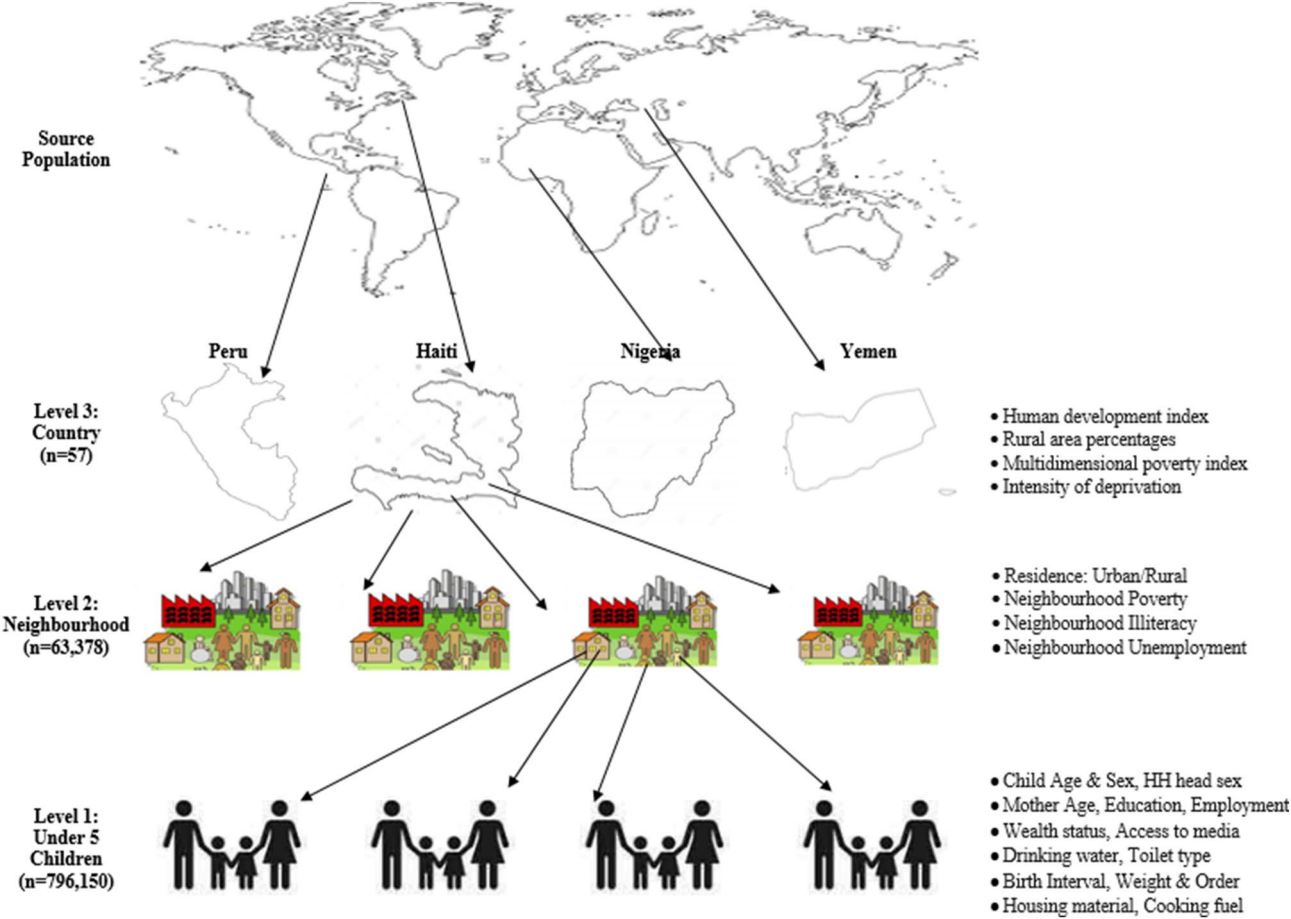
The hierarchical structure of the source data (Authors’ drawings using World Map^39^).

Sex of the children (male versus female), children age (< 12 months (infants) and 12–59 months), household head sex (male or female), mothers’ age (15–24, 25–34, 35–49 years), mothers’ highest education (none, primary, secondary or higher); marital status (never, currently or formerly married), employment status (currently employed or not), access to media (yes or no), sources of drinking water (improved or unimproved), toilet type (improved or unimproved), house building material (improved or unimproved), cooking fuel (clean or unclean), weight at birth (average+, small or very small birth weight), and birth order (1, 2, 3 and 4+). These variables have been linked with diarrhoea in the literature^10,17,20,31,32^. We used the DHS wealth index as a proxy indicator for socioeconomic status. The methods used in computing the DHS wealth index have been described in the literature^38^ as depicted in Fig. 1.

#### Neighbourhood-level factors

In this study, the terms “neighbourhood” and “community”, were used to describe clustering of children within the same geographical living environment^6,40,41^. Neighbours are the children that share the same Primary Sample Unit (PSU) within the DHS data. The PSUs were identified using the most recent census in each country where DHS was carried out^40,42^. The neighbourhood-level factors included in the current study are the place of residence (rural or urban), neighbourhood poverty-, illiteracy- and unemployment levels as illustrated in Fig. 1. The neighbourhood poverty-, illiteracy- and unemployment levels were computed as the proportion of children from households in the lowest two wealth quintiles, children whose mother has no former education and children whose mother was unemployed respectively within each country as of the survey time. We categorized these neighbourhood factors into two levels (low and high) each using the 50th percentile cut-off to allow for non-linear effects and offer useful results for policy decisions. Similar procedures have been used in previous studies^40,42^.

#### Country-level factors

We retrieved the country-level data from the human index reports published by the United Nations database^43,44^. The Human Development Index (HDI) was created by the United Nations to emphasize “that people and their capabilities should be the ultimate criteria for assessing the development of a country, not economic growth alone”^45^. The HDI summarizes the average achievement of countries in three key dimensions of human development: “a long and healthy life, access to knowledge and a decent standard of living”^45^. We categorized the countries into the lowest, middle and highest HDI as shown in Fig. 1. We also explored other country-level factors such as country’s rural area percentages (a measure of the proportion of a countries population that resides in rural areas), multidimensional poverty index (a measure of acute multidimensional poverty) and intensity of deprivation (a measure of the average percentage of deprivation experienced by people in multidimensional poverty)^43–45^. These variables were used for the descriptive statistics but were excluded from the regression models as they correlated with HDI.

### Analytical procedures

We used descriptive statistics to show the distribution of the children by country and by the dependent and independent variables in percentages. Chi-square test of association was used to determine the significance of the association between the independent variables and diarrhoea (Table 1). For the country-level data, we applied sampling weights (SW) provided by the DHS to adjust for unequal cluster sizes, stratifications and to ensure that our findings adequately represent the target population for each country. However, for the pooled data, we computed and applied country-women weights (CWW) to the analysis to reflect the differences in population sizes of the women in each country. The CWW is the product of SW and country-specific weights (CSW). We computed the CSW as the number of sampled women aged 15–49 years divided by the population of women aged 15–49 years for each country. While the number of sampled women is available in the dataset, we obtained the population of each country from United Nations population prospects^46^. We checked multicollinearity among the independent variables using the “colin” command in Stata version 16. The command provided the variance inflation factor (VIF). All variables with VIF > 2.5 were removed from the regression analysis as literature has shown concerns about VIF > 2.5^47^. Statistical significance was set to 0.05. All analysis was conducted in Stata version 16.

**Table 1:** Description of Demographic and Health Surveys data by countries and diarrhoea prevalence among under-five children in LMIC, 2010–2018.

Country	Year of survey	Number of neighbourhoods	Number of under-five children	Prevalence of Diarrhoea (95% CI)
All		63,378	796,150	14.4 (14.2–14.7)
**Eastern Africa**		6298	102,886	16.7 (16.5–16.9)
Burundi	2016	554	12,431	22.5 (21.8–23.3)
Comoros	2012	252	2949	17.0 (15.6–18.3)
Ethiopia	2016	643	9916	11.9 (11.2–12.5)
Kenya	2014	1593	19,889	15.4 (14.9–15.9)
Malawi	2016	850	16,246	21.9 (21.3–22.6)
Mozambique	2011	610	10,157	11.2 (10.6–11.9)
Rwanda	2014	492	7474	12.2 (11.5–13.0)
Tanzania	2015	608	9445	12.1 (11.4–12.8)
Uganda	2016	696	14,379	20.0 (19.4–20.7)
**Middle Africa**		3081	71,630	19.0 (18.7–19.3)
Angola	2016	625	13,463	15.7 (15.1–16.4)
Cameroon	2011	578	10,326	21.7 (20.9–22.5)
Chad	2015	624	16,710	22.3 (21.6–22.9)
Congo	2012	384	8723	19.3 (18.5–20.2)
Congo, DR	2014	536	16,994	17.0 (16.4–17.6)
Gabon	2012	334	5414	16.8 (15.8–17.8)
**Northern Africa**		874	15,458	14.0 (13.5–14.6)
Egypt	2014	874	15,458	14.0 (13.5–14.6)
**Southern Africa**		2544	25,529	15.5 (15.1–16.0)
Lesotho	2014	396	2824	12.2 (11.0–13.4)
Namibia	2013	536	4449	19.1 (17.9–20.2)
South Africa	2016	668	3241	11.0 (9.9–12.1)
Zambia	2018	545	9311	15.5 (14.8–16.2)
Zimbabwe	2015	399	5704	17.1 (16.1–18.0)
**West Africa**		6285	139,382	14.7 (14.5–14.9)
Benin	2018	555	12,512	10.5 (10.0–11.1)
Burkina Faso	2010	573	13,621	14.9 (14.3–15.5)
Coted’Ivoire	2012	351	6876	18.5 (17.5–19.4)
Gambia	2013	281	7633	17.8 (16.9–18.6)
Ghana	2014	427	5539	11.9 (11.0–12.7)
Guinea	2015	401	7213	14.6 (13.8–15.4)
Liberia	2013	322	6806	22.7 (21.7–23.7)
Mali	2018	345	9171	17.2 (16.5–18.0)
Niger	2012	476	11,437	14.4 (13.7–1.05)
Nigeria	2018	1389		12.8 (12.5–13.2)
Senegal	2017	400	11,253	18.0 (17.3–18.8)
Sierra Leone	2013	435	10,254	11.5 (10.9–12.1)
Togo	2013	330	6464	15.2 (14.3–16.1)
**Central Asia**		682	10,216	10.2 (9.6–10.7)
Kyrgyz Republic	2012	316	4222	5.2 (4.5–5.8)
Tajikistan	2017	366	5994	13.3 (12.4–14.1)
**South-Eastern Asia**		1850	17,168	9.0 (8.5–9.4)
Cambodia	2014	609	6934	12.9 (12.1–13.6)
Philippines	2017	1241	10,234	6.1 (5.6–6.6)
**Southern Asia**		33,053	322,219	11.5 (11.4–11.6)
Afghanistan	2015	956	30,520	29.1 (28.6–29.6)
Bangladesh	2014	600	7541	5.7 (5.2–6.2)
India	2016	28,321	247,181	9.2 (9.1–9.3)
Indonesia	2017	1967	17,155	14.2 (13.6–14.7)
Maldives	2016	265	3048	4.2 (3.5–5.0)
Nepal	2016	383	4827	7.7 (6.9–8.4)
Pakistan	2018	561	11,947	19.2 (18.5–19.9)
**Western Asia**		2048	27,441	21.8 (21.3–22.3)
Armenia	2016	306	1709	3.8 (2.9–4.7)
Jordan	2017	962	10,454	9.7 (9.1–10.2)
Yemen	2013	780	15,278	31.4 (30.7–32.1)
**Central America**		1996	22,524	18.7 (18.2–19.2)
Guatemala	2014	856	12,038	19.2 (18.5–19.9)
Honduras	2011	1140	10,486	18.0 (17.2–18.7)
**South America**		1401	9408	12.3 (11.6–13.0)
Peru	2012	1401	9408	12.3 (11.6–13.0)
**Southern Europe**		651	2745	6.1 (5.2–7.0)
Albania	2018	651	2745	6.1 (5.2–7.0)
**Caribbean**		1860	21,129	15.0 (14.5–15.5)
Dominican Republic	2013	516	3560	18.2 (16.9–19.4)
Haiti	2016	449	6082	21.4 (20.3–22.4)
Myanmar	2014	440	4575	10.5 (9.6–11.3)
Timor-Leste	2016	455	6912	10.8 (10.0–11.5)
**Oceania**		755	8415	15.4 (14.6–16.2)
Papua New Guinea	2016	755	8415	15.4 (14.6–16.2)

### Modelling approaches

The multivariable multilevel logistic regression models were used to identify if an association exists between the individual, community contextual factors and national compositional factors and diarrhoea. Using all the 3-level model for binary response specified above, with children *i* who had diarrhoea (at level 1), from a neighbourhood *j* (at level 2), and living in a country *k* (at level 3) as shown in Fig. 1, we identified, constructed and assessed five models to arrive at a robust model that will help identify risk factors of diarrhoea considering the multi-level structure of the data. The models are based on a hierarchical logistic regression model with mixed outcomes consisting of the fixed and random parts as shown in Eq. (1).

1 $$logit\left( {\pi _{{ijk}} } \right) = \underbrace {{\beta _{0} + \sum _{{p = 1}}^{p} \beta _{p} X_{{pijk}} }}_{{Fixed}} + \underbrace {{U_{{0jk}} + V_{{0k}} }}_{{Random}}$$

The probability that a child $i$ of neighbourhood $j$ from country $k$ had diarrhoea is denoted by ${\pi }_{ijk}$. The “*logit*” is the logistic function computed as $logit\left({\pi }_{ijk}\right)=log\left(\frac{{\pi }_{ijk}}{1-{\pi }_{ijk}}\right)$, ${\beta }_{0}$ is the intercept, ${\beta }_{p}$ is the regression coefficient for the $p$ parameters, ${X}_{pijk}$ are the covariates, ${U}_{0jk}$ is the random components due collectively to all children from neighbourhood $j$ of country $k$ while ${V}_{0k}$ is the random components due collectively to all children from country $k$. The mixed model enables detailed exploration of variation in variables between higher-level units (contextual heterogeneity).

We developed five distinct models to enable a detailed assessment of different combinations of factors to select the most robust model that could identify the contextual and compositional risk factors of diarrhoea. This was aimed at modelling the compositional factors and contextual factors separately and collectively, with reference to the distinct multi-level structure of the data used for the analysis. The first model was the null model (Model I) to assess the variation due to the neighbourhood and country-specific random effects without any explanatory variable. It decomposed the magnitude of variance that existed between country and neighbourhood levels. The second model (Model II) included only the individual-level variables conditional on the neighbourhood and country-specific random effects. The third model (Model III) included only the neighbourhood level variables conditional on the neighbourhood and country-specific random effects. The fourth model (Model IV) examined the country-level variables conditional on the neighbourhood and country-specific random effects, while the final model (Model V), estimated the odds of individual, neighbourhood and country-level variables conditional on the neighbourhood and country-specific random effects. All the models were executed using the multilevel regression model of the MLwinN software, version 3.03 embedded in Stata version 15^48^. Parameters were estimated using the Bayesian Markov Chain Monte Carlo (MCMC) procedures^49^ with the following specifications: distribution: binomial; link: logit, burning: 5000, chain: 50,000 and refresh: 500.

### Fixed effects (measures of association)

We reported the results of the fixed effects (measures of association) as the odds ratios (ORs) with their 95% credible intervals (CrIs). Rather than the usual 95% confidence intervals (95% CI) obtained in the frequentist approaches, the Bayesian statistical inference allowed us to summarize probability distributions for measures of association alongside the 95% CrI. The 95% credible interval is simply interpretable as “the 95% probability that the population parameter takes a value in a particular range”.

### Random effects (measures of variation)

In addition to the fixed effects, we also measured the likely effects of the factors considered across the three different levels using the Intraclass Correlation (ICC) and median odds ratio (MOR). The ICC is the measure of the similarity among children living in the same neighbourhood and within the same country. The ICC is a measure of clustering of odds of having diarrhoea in the same neighbourhood and the same country. We calculated the ICC using the linear threshold, which is the latent variable method^50^. Adopting the methods recommended by Larsen et. al. on neighbourhood effects^51^, we reported the random effects in terms of the odds. The MORs are the measures of the variance of the odds ratio in higher levels (neighbourhood and country levels) and it estimates the probability of having diarrhoea that can be attributed to any of the neighbourhood and country factors. If MOR = 1, there is no neighbourhood or country variance. Conversely, the higher the MOR, the more significant are the contextual effects for understanding the probability of developing diarrhoea. A similar approach has been used in similar settings in the literature^52,53^.

### Ethics approval and consent to participate

This study was based on an analysis of secondary data with all identifier information removed. The Institutional Review Board (IRB) of Inner City Fund (ICF) International Macro at Fairfax, Virginia in the USA reviewed and approved the MEASURE Demographic and Health Surveys Project Phase III. The 2010–2018 DHS’s are categorized under that approval. The Institutional Review Board (IRB) of Inner City Fund (ICF) International Macro complied with the United States Department of Health and Human Services Services guidelines and requirements for the “Protection of Human Subjects” (45 CFR 46). All protocols were carried out in accordance with relevant guidelines and regulations on confidentiality, benevolence, non-maleficience and informed consent. All study participants gave written informed consent before participation and all information was collected confidentially. DHS Program has remained consistent with confidentiality and informed consent over the years. ICF Macro ensures compliance with the U.S. Department of Health and Human Services regulations for the respect of human subjects. The authors sought and obtained express approval to use the data from ICF Macro with Accession number 140625. No further approval was required for this study. The data owners can be contacted at thedhsprogram@gmail.com and data can be found at https://www.dhsprogram.com/data/dataset_admin/login_main.cfm. Further documentations on ethical issues relating to the surveys are available at http://dhsprogram.com.

### Patient and public involvement

No patients were involved in the design or dissemination of this analysis.

## Results

### Sample characteristics

In Table 1, we present the distribution of under-five children studied and the weighted prevalence of diarrhoea by the countries, the regions of the world, year of data collection, and the numbers of neighbourhoods per each country. The median number of neighbourhoods per country sampled was 555, ranging from 252 in Comoros to 28,321 in India.

### Measurement of the prevalence of Diarrhoea, special and common cause variations

**Figure 2:**
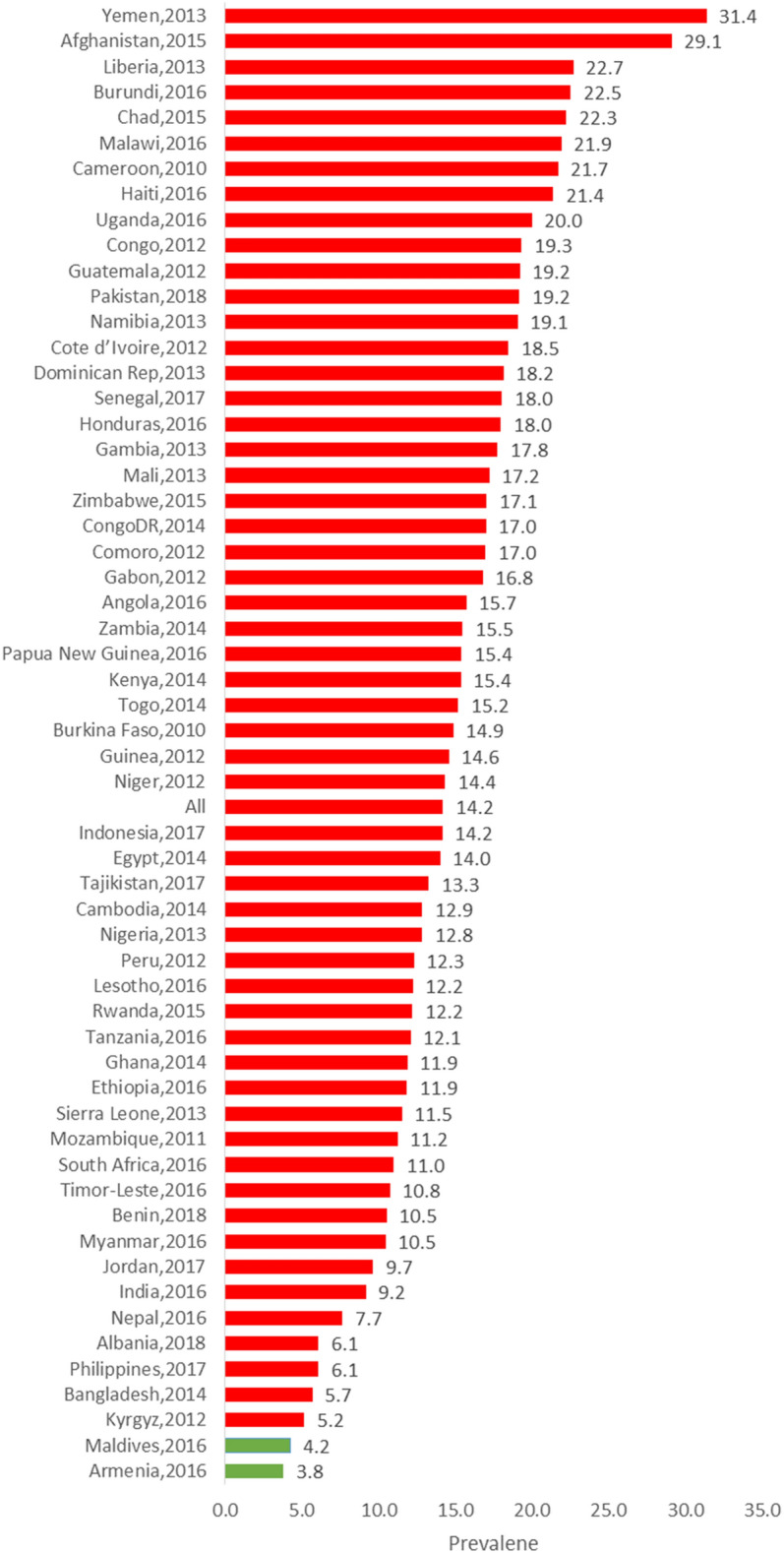
Prevalence of diarrhoea by countries (DHS 2010–2018).

**Figure 3:**
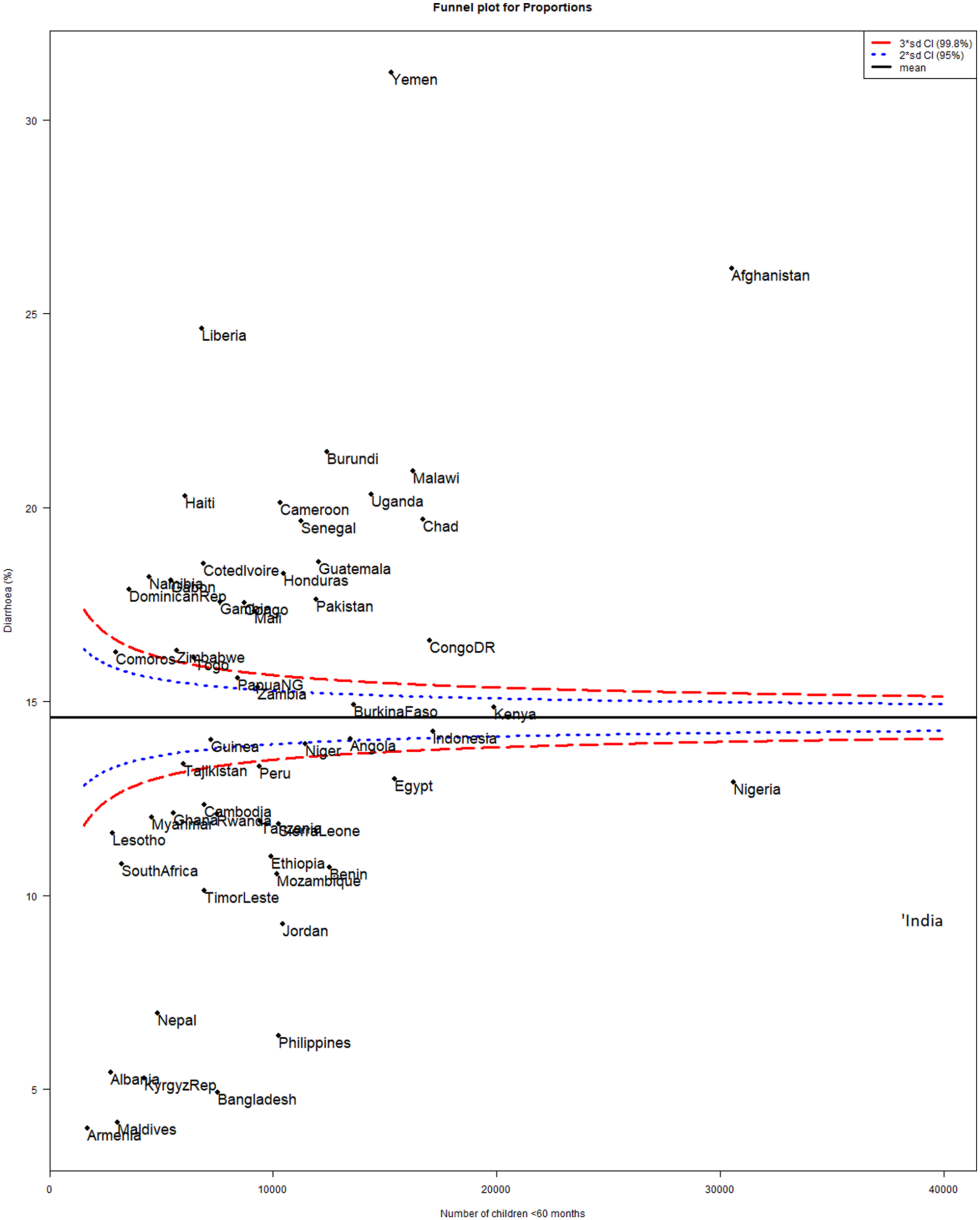
Funnel plot showing common- and special-cause variations in the prevalence of diarrhoea in LMIC (DHS 2010–2018).

As shown in Table 1, Figs. 2 and 3, we found a wide variation in the prevalence of diarrhoea across the countries. The overall prevalence of diarrhoea was 14.4% (95% confidence interval (CI) 14.2–14.7%) ranging from 3.8% (95% CI 2.9–4.7%) in Armenia to 31.4% (95% CI 30.7–32.1%] in Yemen. Considering the regions of the world, the lowest prevalence was found in South Europe (6.1%, 95% CI 5.2–7.0%) while the highest was in Western Asia at 21.8%. The funnel plot in Fig. 3 showed that only 10(17.5%) countries within the 99% control limits, indicating common-cause variation. Twenty-two (38.6%) countries were above the upper control limit and 25 (43.9%) countries were below the lower control limit, indicating special-cause variation (Fig. 3).

### Children individual-level, neighbourhood-level and country-level characteristics

The descriptive statistics by selected individual level, neighbourhood level and country-level characteristics are listed in Table 2. About a fifth (21%) of the children were infants, about half were males (51%) and most of their mothers were aged 25–34 years (52%). A third (32%) of the mothers had no formal education and 43% had at least secondary education while only 17% belong to households in the richest wealth quintiles. Most of the mothers were currently employed (59%) and 81% were from male-headed households. Most (79%) of the children had drinking water from improved sources, only 45% had access to improved toilet types, 72% are from households that use unclean (biomass) cooking gas and only 10% are from a household whose floor, roof and wall materials are all improved.

**Table 2:** Description of background characteristics and diarrhoea prevalence among under-five children in LMIC, DHS 2010–2018.

Characteristics	n	Weighted %	Prevalence of Diarrhoea	X^2 p-value^
**Individual level**				
Age				
Infant	164,438	21.2	16.6 (16.2–17.1)	< 0.001
1 year +	631,712	78.8	13.9 (13.6–14.1)	
Twin				
Single	778,511	97.7	14.4 (14.2–14.7)	
Multiple	17,639	2.3	14.7 (13.5–16.1)	0.7374
Sex				
Female	389,173	49.0	14 (13.7–14.3)	< 0.001
Male	406,977	51.0	14.9 (14.6–15.2)	
Household head				
Male	669,287	80.5	14.5 (14.3–14.8)	0.021
Female	126,863	19.5	14.1 (13.6–14.6)	
Maternal age (years)				
15–24	234,550	27.8	17.5 (17.1–17.9)	< 0.001
25–34	414,014	52.2	13.3 (13.0–13.6)	
35–49	147,586	20.0	13.1 (12.6–13.5)	
Maternal education				
No education	273,056	31.6	16 (15.6–16.4)	< 0.001
Primary	202,835	25.9	16.6 (16.2–17.0)	
Secondary or higher	320,257	42.5	12 (11.6–12.4)	
Wealth Index				
Poorest	202,853	22.5	15 (14.6–15.4)	< 0.001
Poorer	178,258	21.4	14.9 (14.5–15.3)	
Middle	158,228	20.3	14.3 (13.9–14.8)	
Richer	139,713	19.1	14.5 (14.0–15.1)	
Richest	117,098	16.7	13.1 (12.5–13.8)	
Employment				
Employed	526,983	59.3	14.4 (14.1–14.7)	< 0.001
Unemployed	269,167	40.7	14.5 (14.0–14.9)	
No media access				
None	316,993	37.7	15.3 (14.9–15.6)	< 0.001
Yes	478,517	62.3	13.9 (13.6–14.2)	
Drinking water sources				
Unimproved sources	175,663	19.7	16.8 (16.3–17.2)	< 0.001
Improved sources	595,332	80.3	13.9 (13.6–14.2)	
Unimproved toilet type				
Unimproved sources	388,386	44.9	16.2 (15.9–16.5)	< 0.001
Improved sources	382,305	55.1	13 (12.7–13.4)	
Marital status				
Never married	23,560	3.8	18.4 (17.5–19.4)	< 0.001
Currently married	739,740	91.5	14.1 (13.8–14.4)	
Formerly married	32,850	4.6	17.8 (16.9–18.7)	
Cooking fuel				
Unclean/biomass	581,710	72.1	15.7 (15.4–16.0)	< 0.001
Clean fuel	173,921	27.9	11.1 (10.6–11.6)	
Housing materials				
Unimproved sources	676,227	89.8	14.9 (14.6–15.2)	< 0.001
Improved sources	79,157	10.2	10.2 (9.7–10.8)	
Weight at birth				
Average +	643,472	82.0	14.2 (13.9–14.4)	< 0.001
Small	90,809	13.4	15.7 (15.1–16.3)	
Very small	31,924	4.7	19 (18.1–20.0)	
Birth interval				
1st birth	223,779	28.0	13.7 (13.3–14.1)	< 0.001
< 36 months	308,310	37.0	15.4 (15.0–15.7)	
36 + months	262,278	35.0	14.1 (13.7–14.4)	
Birth order				
1st	223,777	27.9	13.7 (13.3–14.1)	< 0.001
2nd	192,088	23.7	13.4 (13.0–13.8)	
3rd	129,829	16.4	14.2 (13.7–14.7)	
4 +	250,456	32.0	16 (15.7–16.4)	
**Neighbourhood level**				
Location				
Urban	239,222	34.0	14.1 (13.6–14.7)	< 0.001
Rural	556,928	66.0	14.6 (14.3–14.9)	
Neighbourhood SES				
Highest	159,709	13.5	11.1 (10.6–11.7)	< 0.001
2	158,969	24.5	14.8 (14.1–15.5)	
3	160,077	23.6	15.1 (14.6–15.7)	
4	159,153	23.5	15.4 (14.8–15.9)	
Lowest	158,242	14.9	14.3 (13.8–14.9)	
Community poverty rate				
Low	398,524	50.6	14.2 (13.9–14.6)	< 0.001
High	397,626	49.4	14.7 (14.3–15.0)	
Community illiteracy rate				
Low	393,382	50.0	14.4 (14.0–14.7)	< 0.001
High	402,768	50.0	14.5 (14.2–14.9)	
Community unemployment rate				
Low	273,610	42.6	15.6 (15.2–16.1)	< 0.001
High	522,540	57.4	13.5 (13.2–13.9)	
**Country level**				
Deprivation intensity				
Low deprivation	252,671	40.8	14.9 (14.5–15.4)	< 0.001
High deprivation	543,479	59.2	14.1 (13.8–14.4)	
Human development Index				
lowest	319,367	43.9	18.5 (18.1–18.9)	< 0.001
Middle	440,445	42.4	12.3 (11.9–12.6)	
Highest	36,338	13.7	8.2 (7.5–9.0)	
Rural percent				
Low rural %	172,261	24.0	16.3 (15.8–16.8)	< 0.001
High rural %	623,889	76.0	13.8 (13.5–14.1)	
Multidimensional poverty				
Low MDPI	579,233	56.3	12.8(12.5–13.1)	< 0.001
High MDPI	216,917	43.7	16.5(16.1–17.0)	
Total	796,150	100.0	14.4(14.2–14.7)	

On the neighbourhood-level factors, 66% of the children lived in rural areas, 49% from communities with high poverty rate, 50% and 57% were from communities with high illiteracy rate, and high unemployment rate respectively. Three-fifths (59%) of the children are from countries with a high level of intensity of deprivation and 44%, 42% and 14% from countries with the lowest, middle and high HDI respectively. All the variables considered at the individual-, neighbourhood- and county-levels were significantly associated with diarrhoea in a Chi-square test and the bivariate logistic regression models between each of the explanatory variables and diarrhoea. Hence, all the variables were candidates in the multivariable models.

### Measures of associations (fixed effects) of having Diarrhoea

Table 3 presents the outputs of each of the different models explored in this study. In the fully adjusted model (Model V) wherein we controlled for the effects of the individual-, neighbourhood- and country-level factors, children age, children sex, mothers educational attainment, mothers age, employment status, media access, sources of drinking water, toilet type, marital status, housing material, cooking fuel type, weight at birth, birth order, place of residence (rural or urban), neighbourhood poverty-, illiteracy- and unemployment rates, as well as HDI were significantly associated with odds of diarrhoea.

**Table 3:** Individual, neighbourhood and country factors associated with the diarrhoea identified by multivariable multilevel logistic regression models, DHS data, 2010–2018.

Characteristics	^a^Model I	^b^Model II	^c^Model III	^d^Model IV	^e^Model V
	aOR (95% CrI)	aOR (95% CrI)	aOR (95% CrI)	aOR (95% CrI)	aOR (95% CrI)
**Fixed-effect**					
Individual-level					
Infants vs 1–5 year children		1.29 (1.26–1.31)			1.29 (1.26–1.31)
Male child (vs female)		1.11 (1.09–1.12)			1.11 (1.09–1.12)
Female Household head vs male		1.01 (0.99–1.03)			1.01 (0.99–1.03)
**Maternal age (years)**					
15–24		1.70 (1.66–1.75)			1.70 (1.65–1.75)
25–34		1.23 (1.21–1.26)			1.23 (1.20–1.26)
35–49		Reference			
**Maternal education**					
No education		0.96 (0.94–0.99)			0.95 (0.92–0.97)
Primary		1.05 (1.02–1.07)			1.04 (1.02–1.06)
Secondary or higher		Reference			
**Wealth index**					
Poorest		1.22 (1.17–1.26)			1.23 (1.18–1.27)
Poorer		1.18 (1.14–1.22)			1.19 (1.15–1.24)
Middle		1.15 (1.12–1.19)			1.16 (1.13–1.20)
Richer		1.12 (1.09–1.15)			1.12 (1.09–1.15)
Richest		Reference			
Unemployed (vs employed)		1.09 (1.07–1.11)			1.09 (1.07–1.11)
No media access(vs access)		1.04 (1.02–1.06)			1.04 (1.02–1.06)
Unimproved drinking water		1.02 (1.00–1.05)			1.02 (1.00–1.05)
Unimproved toilet type		1.04 (1.02–1.06)			1.04 (1.02–1.06)
**Marital status**					
Currently married		Reference			
Never married		1.07 (1.02–1.12)			1.07 (1.03–1.12)
Formerly married		1.10 (1.06–1.14)			1.10 (1.06–1.14)
Clean cooking fuel vs biomass		1.02 (1.00–1.05)			1.02 (0.99–1.05)
Improved housings vs unimproved		1.04 (1.01–1.07)			1.04 (1.01–1.07)
**Weight at birth**					
Average +		Reference			
Small		1.18 (1.16–1.21)			1.18 (1.16–1.21)
Very small		1.37 (1.33–1.42)			1.37 (1.32–1.42)
**Birth order**					
1st		Reference			
2nd		1.08 (1.06–1.10)			1.08 (1.06–1.10)
3rd		1.18 (1.15–1.21)			1.18 (1.15–1.21)
4+		1.32 (1.29–1.36)			1.32 (1.29–1.36)
**Neighbourhood-level factor**					
Rural v urban			0.99 (0.96–1.01)		1.05 (1.03–1.08)
High v low poverty rate			1.08 (1.05–1.10)		1.01 (0.99–1.04)
High v low illiteracy rate			1.07 (1.04–1.10)		1.07 (1.04–1.10)
High v low unemployment			0.98 (0.95–1.00)		1.00 (0.97–1.02)
Country-level factor					
Human development index (upper)					
Lowest				2.44(1.82–3.33)	2.66 (1.65–3.89)
Middle				1.78(1.29–2.33)	1.88 (1.23–2.83)
**Random effects**					
Country-level					
Variance (95% CrI)	0.30 (0.21–0.44)	0.28 (0.19–0.41)	0.31 (0.21–0.45)	0.23(0.16–0.34)	0.22 (0.15–0.33)
VPC (%, 95% CI)	7.36 (5.15–10.3)	6.83 (4.78–9.63)	7.54 (5.28–10.6)	5.71(3.96–8.12)	5.56 (3.83–7.92)
MOR (95% CrI)	1.69 (1.54–1.89)	1.65 (1.51–1.84)	1.7 (1.55–1.9)	1.58(1.46–1.74)	1.57 (1.45–1.73)
Neighbourhood-level					
Variance (95% CrI)	0.51 (0.49–0.53)	0.50 (0.49–0.52)	0.51 (0.49–0.53)	0.51(0.49–0.53)	0.50 (0.49–0.52)
VPC (%, 95% CI)	19.8 (17.5–22.8)	19.2 (17.1–22.0)	19.9 (17.6–22.9)	18.4(16.5–20.8)	18.1 (16.2–20.5)
MOR (95% CrI)	1.98 (1.96–2.00)	1.97 (1.95–1.99)	1.98 (1.95–2.00)	1.98(1.96–2.00)	1.97 (1.95–1.99)
**Model fit statistics**					
Bayesian DIC	588,993	546,557	588,887	588,987	545,122
Sample size					
Country-level	57	57	57	57	57
Neighbourhood-level	63,378	62,156	63,378	63,378	62,156
Individual-level	796,150	751,837	796,150	796,150	751,837

The OR in bold suggest significance at 5%

*OR* odds ratio, *CrI* credible interval, *MOR* median odds ratio, *VPC* variance partition coefficient, *DIC* Deviance Information Criteria.

^a^Model I—empty null model, baseline model without any explanatory variables (unconditional model).

^b^Model II—adjusted for only individual-level factors.

^c^Model III—adjusted for only neighbourhood-level factors.

^d^Model IV—adjusted for only country-level factors.

^e^Model V—adjusted for individual-, neighbourhood-, and country-level factors (full model).

The adjusted odds of diarrhoea was 29% higher among infants than those aged 12–59 months (adjusted odds ratio (aOR) = 1.29, 95% CrI 1.26–1.31). Male children were more likely to have diarrhoea (aOR = 1.11, 95% CrI 1.09–1.12). The odds of diarrhoea was also higher among children whose mothers were aged 15–24 and 25–34 years compared with children whose mothers were aged 35–49 years (15–24: aOR = 1.70; 95% CrI 1.65–1.75 and 25–34: aOR = 1.235; 95% CrI 1.20–1.25). The odds of diarrhoea was significantly less likely among children with no maternal education than the children whose mothers have at least secondary education (aOR = 0.95; 95% CrI 0.92–0.97). Children from households in the poorest wealth quintiles were 23% more likely to have diarrhoea than those from households in the uppermost wealth quintiles (aOR = 1.23, 95% CrI 1.18–1.27). Children whose mothers were unemployed had higher (9%) odds of diarrhoea (aOR = 1.09; 95% CrI 1.07–1.11). Inaccessibility to media increased the odds of diarrhoea by 4% (aOR = 1.04; 95% CrI 1.02–1.06). The odds of diarrhoea was 37% and 18% higher among children who had very small (aOR = 1.37; 95% CrI 1.32–1.42) and small (aOR = 1.18; 95% CrI 1.16–1.21) birth weight respectively than those that had average or bigger birth weights. The odds of diarrhoea was 5% higher in rural areas than in urban areas (aOR 1.05; 95% CrI 1.03–1.08). Children from neighbourhoods with high illiteracy (aOR = 1.07, 95% CrI 1.04–1.10) rates were more likely to have diarrhoea than those from neighbourhoods with low illiteracy rate. At the country-level, the odds of diarrhoea nearly doubled (aOR = 1.88, 95% CrI 1.23–2.83) and tripled (aOR = 2.66, 95% CrI 1.65–3.89) among children from countries with middle and lowest HDI respectively compared with those from highest HDI countries.

### Measures of variations (random effects) of having Diarrhoea

Model I (the null model), showed that there was a significant variation in the odds of developing diarrhoea across the countries (σ^2^ = 0.30, 95% CrI 0.21–0.44) and across the neighbourhoods (σ^2^ = 0.51, 95% CrI 0.49–0.53). On the assessment of the intra-country and intra-neighbourhood correlation coefficient, 7.4% and 19.8% of the variance in odds of having diarrhoea could be attributed to the country- and neighbourhood-level factors, respectively. The median odds ratio (MOR) in the nested model (Model V) confirmed evidence of neighbourhood (1.57) and societal contextual (1.97) phenomena shaping the distribution of diarrhoea among under-five children as shown in Table 3. Model V revealed significant variation in the odds of developing diarrhoea across both the neighbourhoods (σ^2^ = 0.50, 95% CrI 0.49–0.52) and the countries (σ^2^ = 0.22, 95% CrI 0.15–0.33). Going by Model V, 6% of all variability in having diarrhoea was explained by the countries from which the children live compared with 22% explained by their neighbourhood differences.

## Discussion

Using the information provided by parents and guardians of 796,150 under-five children from 57 LMIC, we explored the factors associated with the experience of at least one episode of diarrhoea within 2 weeks preceding the survey dates in each of the countries. The proportion of children who experienced diarrhoea varied widely across the 57 countries from 4% in Armenia to 29% in Afghanistan. Our major finding is that factors that predispose children to diarrhoea are diverse and complex. The factors are made up of individual cum household, neighbourhood and country-level factors. These characteristics formed distinct blocks of compositional and contextual factors associated with diarrhoea. The compositional factors include being an infant, males, from female-headed households, mother aged < 35 years, mother had primary education, unemployed, mother never married, from a household in the lower wealth quintiles, and no media access to be at higher odds of diarrhoea. Other significant compositional factors include drinking water from unimproved sources, uses unimproved toilet types, small weight at birth, high birth orders. The contextual factors are residing in rural areas, from communities with high poverty, illiteracy and unemployment rates and from countries with the lowest and middle HDI.

We found diarrhoea episodes to be commoner among infants than the older under-five children. This is consistent with existing findings in the literature^1,17,23,31,32,54^ and could be attributed to more fragile anatomy of infants as well as the exclusiveness of breastfeeding^14^. Particular attention should be paid to the prevention of diarrhoea among infants as the higher cases among them has been linked with higher fatalities than among the older children^55^. We also found higher odds of having diarrhoea among male children compared with their female counterparts. Similar differences have been identified in the literature^23^ but at variance to the findings of Tetteh et al. that diarrhoea was higher among female children^56^.

The odds of having diarrhoea reduced with increments in mothers’ age. The odds were higher among children whose mothers were aged 15–24 years and 25–34 years compared with those born to women aged 35–49 years. Similar findings have been reported in the literature^57,58^. These differences may not be unconnected with the fact that teenage and young adult motherhood comes with its challenges including neglect, limited resources and the likelihood of contracting diseases by both the young mothers and their children^59^. Also, it is not unlikely that older mothers are more experienced in preventing diarrhoea among under-five children. Therefore, age-specific intervention could be designed to prioritise the younger mothers.

Educational attainment among mothers has been associated with childhood diseases including diarrhoea^60^. Our findings generally suggested that children from mothers with limited educational attainment are more likely to have diarrhoea as corroborated in the literature^10,30,32,57,60,61^. The differences were more distinct among children whose mothers had only primary and those that had secondary or higher education. This is a clear indication that other factors interact with women education in the likelihood of children having diarrhoea. Education alone may be insufficient in preventing diarrhoea, factors such as household wealth status, access to media, hygiene and sanitation, good water, rural–urban residential, women age etc. are also important in the prevention of diarrhoea. For instance, higher educational attainment is associated with a better awareness of health education including knowledge and guidelines on sanitation, hygiene, feeding and weaning practices etc^60^.

The wealth status of the households to which the children belong appeared to have played a dominant and consistent role in whether a child experience diarrhoea or not across the LMIC studied. Our findings are in agreement with earlier reports^31,60,61^. There were linear increments in the odds of having diarrhoea from those in households in the poorest wealth quintile compared to those in the richest wealth quintile. The likelihood of diarrhoea was generally 23% higher among children from households in the poorest wealth quintiles than those in the richest wealth quintile. The role of wealth, or at least purchasing power, in the knowledge and utilization of health care services, and by extension, in health outcomes, have been documented^62^. Fagbamigbe et al. reported that women from a household in higher wealth quintiles have a higher likelihood of health care utilization in Nigeria^62^. Wealth is a vital tool in gaining access to media, good sanitation and hygiene, clean cooking fuel etc. To prevent diarrhoea in LMIC, there is a need to enhance the means of livelihood and alleviate poverty among mothers generally since most people in these countries currently live below $2 per day^43^. Livelihood enhancement and poverty alleviation strategies could include employments and better education.

In the current study, we identified access to improved sources of drinking water, use of improved toilet types, use of improved housing materials (floor, wall and roof) and use of clean cooking fuel in households to have lowered the odds of diarrhoea in LMIC. As noted by Fagbamigbe et al., poor hygiene and sanitation including the use of unimproved toilets and water sources have a direct pathway to diarrhoea^20^. We could not assess the effect of “use of soap for hand hand-washing before meals and meals preparation” in this study because the information was not available for most countries. Nonetheless, our result is corroborated with findings from other studies, where diarrhoea have been linked with hygiene, water and sanitation^1,10,54,60,63,64^. Adequate practice and maintenance of good sanitation, hygiene etc. can reduce the risk of diarrhoea. Efforts should be made to enhance the knowledge and capacity of women and households, in general, to maintain good sanitation and hygiene in addition to the use of improved housing materials and access to safe drinking water.

Health promotion and education on the prevention of diarrhoea are often disseminated through media such as radio, television and newspaper. Access to media on diarrhoea prevention has an indirect link to diarrhoea occurrences. Media access improves knowledge about diarrhoea, which in turn enhances preventive and management practices^65^. We identified that the children whose mothers had no access to at least one of these media sources had higher odds of developing diarrhoea. This finding is consistent with what has been reported in the literature^22,66^. However, access to media could be limited by educational attainment, household wealth status and availability of social infrastructures such as electricity which is lacking in most households and communities across the LMIC. Besides media, there may be a need to reach the mothers directly through local postnatal providers and peer education.

Also, children with low birth weights had higher odds of developing diarrhoea compared with those that normal birth weights as reported by Bado et al.^54^. Greater attention should be paid to the health needs and challenges of children with low birth weights to reduce their chances of developing diarrhoea and other childhood diseases. Children with low birth weight are more susceptible to morbidities and mortality. Therefore, it has a causal pathway to diarrhoea. Using birth order as a proxy for the current family size, we found that the odds of having diarrhoea increased consistently with the increase in the birth order of the children. The prevalence rose from 8 to 18% to 32% among those with 2nd, 3rd and 4th or higher birth orders respectively compared with the children who were first births. Similar findings that diarrhoea is commoner among children in large households have been reported^1,61^. This is plausible as larger households can overstretch the limited resources at their disposals. More so, larger family size has been reported to be commoner among households in lower wealth quintiles^67^. This further corroborates our finding on the association between poverty and diarrhoea.

On the contextual factors, we found higher odds of diarrhoea in the rural area compared with the urban areas as reported previously^66,68^ but at variance with an Ethiopian study which reported higher odds in urban areas^10^. Also, children from communities with high deprivations in terms of high poverty, illiteracy and unemployment rates had a higher likelihood of experiencing diarrhoea episode compared with the other children from advantaged communities^10,69^. These contradictions could be ascribed to the specifics of each rural and urban areas. For instance, Kenya has a large slum within its capital city, Nairobi. Diarrhoea experience in such slums with high population density within urban areas could be higher than in rural areas with better and cleaner natural sources^70,71^.

In a similar pattern, children from countries with the lowest and middle HDI have higher odds of having diarrhoea than those from countries with the highest HDI. Of all the factors considered in this study, countries’ HDI levels presented the highest odds of diarrhoea. While the odds of diarrhoea nearly doubled among children from countries in the middle HDI, it nearly tripled among those from countries having the lowest HDI. This clearly showed that there are country-level contextual factors and other compositional factors that predispose children to diarrhoea. Our finding aligns with previous findings of Mokomane et al. and Ahs et al.^17,22^.

Our findings provide evidence of wide variations in the development of diarrhoea within and across the LMIC. The dis-advantaged communities (those with a high rate of unemployment, illiteracy, poverty) and countries (those lowest human capital development index) are the worst hit by diarrhoea. Efforts should be made to increase the overall well-being of every community as children from more deprived communities, irrespective of the differences in their compositional factors, all have higher odds of having diarrhoea than their peers from better-off communities. Enhancing the development of LMIC in all spheres will sustain human progress, reduce vulnerabilities and build resilience. As pointed out in earlier reports, there are needs for efficient and effective interventions to guide strategies to target risk factors unique to communities and countries^14^. The implications of the findings of this study for clinical practices is that clinical practices alone may be insufficient in reducing diarrhoea incidences. Besides adequate platform to manage diarrhoea cases clinically, mothers’ and community-level characteristics should be considered in designing strategies to reduce diarrhoea episodes among children. The identified contextual and compositional factors in this study are “modifiable” as far as diarrhoea preventive interventions are concerned. Through appropriate intervention, the factors could be explored as a means of reducing the occurrence of diarrhoea among under-five children in LMIC.

### Study limitation

The data used for this study relied on mothers and guardians/caregivers recall of diarrhoea episodes among their under-five children. This might have introduced a recall bias through underreporting or over-reporting of the cases. However, DHS has incorporated check and control mechanisms to ensure the accuracy of data collected across the countries. Therefore, the recall bias posed no threat to the reliability of our estimates. The cross-sectional nature of the data prevented causal inferences. Nonetheless, the associations established with the risk factors is suitable to design intervention strategies. Also, the secondary nature of the data has limited our choice of community-level independent factors but we were able to generate quality community-level variables to identify the contextual factors. Besides, we have used only quantitative data, availability of qualitative data could have helped dissected the contextual and compositional factors better. These limitations could be addressed by collecting primary data that includes both quantitative and qualitative data. The use of nationally representative data with proven data reliability and integrity have given credence to the reliability of our findings. The strength of our study lies in its ability to pool the diarrhoea experience of about three-quarters of a million children from 57 countries to arrive at our estimates and conclusions.

## Conclusion

Diarrhoea remains a major problem in most LMIC studied. We identified diverse individual-level, community-level and national-level factors associated with the development of diarrhoea among under-five children in these countries. In all, we found the highest odds of diarrhoea among the poorest children from the less-advantaged communities within countries with the lowest human development index. Thus, there is a need to reduce the incidence and prevalence of diarrhoea among under-5 year children to forestall a possible/likely rebound in the upsurge of diarrhoea-associated mortality in the nearest future.

### Recommendations

There is a need to reinforce diarrhoea prevention and control program at all levels-community, national and global—across the low and middle-income countries to reduce the chances of an under-five child developing diarrhoea. In particular, interventions should include community-level health education and promotion on ways to avert diarrhoea incidences are the best measures to reduce its occurrences. Poverty alleviation through gainful employment and better education among women remains the gateway to necessary information on strategies to guide against diarrhoea. To achieve a meaningful reduction in the prevalence of diarrhoea, there may be a need to involve community and religion leaders to influence communal behaviour and practices that could enhance overall community sanitation.

## Data Availability

The data supporting this article is available at http://dhsprogram.com on request from the owners of the data.

## Abbreviations

CI: Confidence interval
CrI: Credible interval
CSW: Country specific weights
CWW: Country-women weights
DHS: Demographic and health survey
GBD: Global burden of disease
HDI: Human development index
ICC: Intraclass correlation
ICF: Inner city fund
IRB: Institutional Review Board
LMIC: Low- and middle-income countries
MCMC: Markov chain Monte Carlo
MOR: Median odds ratio
PSU: Primary sample unit
SES: Socioeconomic status
SDGs: Sustainable development goals
SW: Sampling weights
UNICEF: United Nations International Children’s Emergency Fund
VIF: Variance inflation factor
WHO: World Health Organization
